# Revisiting Environmental Belief and Behavior Among Ethnic Groups in the U.S.

**DOI:** 10.3389/fpsyg.2019.00629

**Published:** 2019-03-27

**Authors:** Vincent Medina, Alyssa DeRonda, Naquan Ross, Daniel Curtin, Fanli Jia

**Affiliations:** Department of Psychology, Seton Hall University, South Orange, NJ, United States

**Keywords:** ethnicity, environmental belief, environmental behavior, USA, ethnic minority, immigrant

Maslow's hierarchy of needs (1970) depicts a simple, five-part pyramid with fundamental needs on the bottom and secondary needs near the top. The environmental hierarchy of needs theory, which pulls from Maslow's hierarchy, has commonly been used to suggest that ethnic groups hold less environmental concern and action than their White counterparts (Van Liere and Dunlap, 1980; Taylor, 1989; Mohai, 1990; Sheppard, 1995). The logic is as follows: sociological demographics suggest that minority populations tend to have lesser wealth and education. Therefore, minorities are more likely to focus on physiological needs necessary for survival, and in turn generally have less time and resources to allocate toward other problems. Environmental protection naturally becomes a secondary concern. This style of thinking was first popularized in the 1970s, with one widely cited study conducted by Hershey and Hill (1977). They found that there was a gap between White and African American students on their concerns for the environment. However, many of the cross-ethnic environmental studies conducted in the following decades have produced highly conflicting evidence with regard to the conceptualization of pro-environmental behaviors in different ethnic groups (for a review, see Head et al., 2018). In this article, we first review past studies on environmental belief and behavior selectively from both national surveys and regional representative samples (excluding convenience samples), paying attention to the emergence of ethnicity. These studies generated inconsistent answers to the question of how ethnic minorities respond to the environmental issue. Then, we argue that past studies overestimated the individual level of analysis, such as individual norms and beliefs, but underestimated the power of contextual analysis such as group norms, cultural orientations, and economic factors. We support our viewpoint by identifying conceptual and methodological issues that are important to consider for future research.

## Selective Reviews on Past Literatures

Environmental behavior varies significantly between ethnic groups. On the one hand, past studies have shown that ethnic minorities in the United States engage in fewer pro-environmental behaviors. This view was shown in a study by Johnson et al. (2004), which thoroughly examined environmental beliefs and action in the National Survey on Environment. They found that when age, gender, education, residence, and political views were all controlled for, African Americans and foreign-born Latinos scored significantly lower on environmental belief [a questionnaire from the New Ecological Paradigm (NEP)] and on four environmental behaviors (recycling, environmental reading, nature participation, and joining of conservation groups) than their White counterparts. A similar result indicated that African Americans scored significantly lower than European Americans on an index of environmental behavior in a sample of 720 residents in Detroit, Michigan (Parker and McDonough, 1999). In addition, African Americans in the public sample showed less concern for both chemical and global risks (e.g., ozone depletion and global warming). These results are consistent with prior literature regarding ethnicity and environmentalism (Kalof et al., 2002; Slimak and Dietz, 2006).

On the other hand, several recent studies have revealed that non-whites in the U.S. reported greater concerns and engagements about the environment (e.g., climate change and protection of the environment) than Whites (c.f. Pearson et al., 2017). For an example, Whittaker et al. (2005) compiled over two decades of California Field Polls (with the majority of answers coming from Whites, African Americans, and Latinos) with regard to various environmental issues: pollution concern, environmental protection concern, toxic waste concern, increased taxes for environmental regulation, self-identified environmentalist, and opposition to offshore drilling. They found that African Americans and Latinos have been concerning and engaging in more pro-environmental behaviors than White Americans over time except on the topic of offshore drilling after controlling for socioeconomic status, education, age, and gender. Similarly, studies on Gallup polls between 2001 and 2010 (McCright and Dunlap, 2011), and a 2014 national representative sample (Jones et al., 2014) found that non-Whites reported greater concerns about global warming, climate change, and environmental threats to personal lifestyles than Whites. In addition, Macias (2016) investigated environmental risk perception across nine ethnic groups in the U.S. by using the 2010 General Social Survey and pulled from ~1,500 responses across the nation. They concluded Mexican and Latin American immigrants, as well as African Americans, held a high threat perception of air pollution, nuclear power plants, and climate change.

## Suggestions for Future Studies

So where does this leave us? As mentioned earlier, a significant limitation in environmental psychology is that there is an inadequate amount of studies conducted with ethnic minority groups. As of now, the literature presents conflicting results regarding the level of environmental concern and pro-environmental behavior within ethnic minority groups. Additional research is needed to resolve the conflicting positions and propose a new perspective on ethnic minorities' environmental attitude and engagement. Therefore, future research should consider different conceptualizations and methodologies regarding environmental beliefs and actions among ethnic minorities.

However, past research on the topic has primarily emphasized individual beliefs toward environmental issues and underestimated the role of social contexts in which members of ethnic minority groups live. Bronfenbrenner's (1979) ecological model has indicated that behavioral development is influenced by interacting with various contexts such as *microsystems* (e.g., individual beliefs), *exosystem* (e.g., local economics), and *macrosystem* (e.g., cultural orientations). In Bronfenbrenner's view, these systems are interrelated, which means that behaviors occurring at the smallest level of context can be influenced by what occurs in the largest context. In other words, individual pro-environmental behaviors are affected by both individual norms, and the norms and expectations within their social contexts (e.g., in-group social norms, local economics, and diverse cultural orientations). In the following sections, we discuss the evidence and suggest that both individual and contextual factors play important roles regarding ethnic groups' attitude and behavior on environmental issues. In the end, we offer a mixed-method approach to synthesize both individual and contextual factors in future empirical studies.

## Integrating both Individual and Social Norms in Value-Belief-Norm Theory

Conceptually, previous studies investigating ethnic differences in environmental concerns were largely based on value-belief-norm theory (VBN) (Stern and Dietz, 1994). The theory combines three components (i.e., personal norms, values, and environmental beliefs) into a holistic view of environmental behavior (Stern, 2000; Milfont and Page, 2013). The VBN theory has been shown to predict consumers' decision-making, ecological behavior, environmental policy, and awareness of the consequences of environmental actions (Dietz et al., 2005). However, past environmental research among American ethnic groups have largely focused on the segment of environmental beliefs via the NEP (e.g., Bechtel et al., 1999; Corral-Verdugo and Armendariz, 2000; Schultz et al., 2000; Rauwald and Moore, 2002; Johnson et al., 2004). Despite widespread use of the NEP, recent research suggests that it may be inappropriate for use in diverse populations (Klain et al., 2017). The NEP lacks cultural context because it was designed within a Western individualistic framework. As opposed to a collectivistic framework that places values on the relationships. This is why, for example, the NEP is inappropriate for research use in India: there is a vast East-West difference in traditions, worldviews, and different sociodemographic variables (Chatterjee, 2008).

This VBN framework to environmental behaviors may be influenced by in-group social norms across demographic groups in addition to personal values and beliefs. In-group social norms refer to what other people are doing or what people should do in the context of family, friend, community, and other in-group members (Schultz et al., 2007). A recent study has argued that the in-group social norms may help explain different levels of engagement between minority groups (Ballew et al., 2019) because members in minority groups have valued the needs and goals of in-group more importantly than Whites. Indeed, researchers found that in-group social norms (friends and family are taking environmental actions) positively predicted Latino Americans' environmental engagement (Ballew et al., 2019). A similar result showed that minority groups reported greater concerns on climate change and willing to engage in environmental advocacy than Whites when environmental issues have direct impacts on their local communities (Pearson et al., 2017).

In addition, Eom et al. (2018) proposed that if personal values and norms are not strong factors of environmental actions, in-group social norms should motivate people to act environmentally, especially with a low SES background. In their experiments, they found personal beliefs about climate change predicted pro-environmental action (probability of donation for sustainability) only in a high SES group. However, social norms (beliefs about their close others such as family, friends, and classmates at the same university) intentions to act pro-environmentally positively predicted the probability of donation in a low SES group.

However, research on “in-group social norms” may serve a stereotypical view of “non-environmentalist” among minority groups. Pearson et al. (2018) pointed out that pro-environmental activities may not be viewed as simply personal choices made individually but rather are perceived as in-group social norms. If pro-environmental activities are supposed as a part of White middle-class social norms, then ethnic minorities members who are not in the “in-group White middle-class” category would be misperceived stereotypically incongruent of their social identities (e.g., as non-environmentalists). For example, Pearson et al. (2018) found the term “environmentalist” was more positively associated with White than with other minority groups. This misperception of being non-environmentalists as a “social norm” in minority groups predicted the minority group's low level of environmental concern, indicating that social norms are inferred through the stereotypical perception of non-environmentalists in minority groups. Taking together, future research should investigate the VBN in a holistic approach by studying multiple determinants including both micro levels (e.g., personal values) and macro levels (e.g., social norms and local communities) simultaneously across different minority groups.

## Considering Diverse Cultural Orientations in the U.S

There is a dichotomous focus on White American vs. African American or Latino American differences while disregarding the increasing diversity in the U.S. population from different cultural orientations (e.g., Asian Americans and Native Americans). Little research is available about the impact of various Eastern cultures on the natural environment, even though past literature has revealed cross-cultural differences on environmental behaviors and perception of environmental concerns across different societies (see Milfont, 2012; Milfont and Schultz, 2016). For example, Eom et al. (2016) found that the common logic that concerns-about the environment lead to pro-environmental action is more applicable to Western cultures than to Eastern cultures when comparing results from 47 nations. In addition, certain countries tend to be more resource conscientious than others. When the countries of the world are measured by negative environmental impact (e.g., carbon emissions, endangered species, habitat loss, water pollution), there are distinct ranks, with Asian countries having the most room for improvement (Bradshaw et al., 2010). However, it is important to keep in mind that this rank is not likely entirely reflective of Eastern cultures concern toward the environment. It only measures negative environmental impact, which could be attributed to external factors such as Asian countries' large population and resource requirements or limited availability of clean energy technology.

In addition, research on environmental values and beliefs within indigenous cultures (e.g., Native Americans) have been scarce. A recent study by Washinawatok et al. (2017) explored Native American children's (rural Menominee and urban Native Americans in Chicago) understanding of the natural environment with a unique measure, a 3-dimensional diorama with real models of trees, water, grass, and rock to provide a context in which to interact with toy animals. It is worth noting that a Native American research member rejected the traditional way of measuring human-animal interaction (with plastic toy animals in hypothetical situations) because Native American children would view the plastic animals alone as unnatural and ecologically inappropriate on perceiving nature. They found that Native American children were significantly more interested in playing with the diorama than playing with the toy animals, and were more likely than non-Native American children to engage in perspective taking within nature environment (Washinawatok et al., 2017). In a similar line of research, Cowie et al. (2016) examined the environmental values of indigenous people's (Maori) in New Zealand. They found that the Maori people expressed higher levels of environmental values than European New Zealanders partially due to Maori people's high sociopolitical consciousness. Therefore, historical contexts, economic dynamics, and political orientations need to be considered among the indigenous population in environmental research (Clark, 2002).

Another related, understudied topic of interest to consider is immigrant environmental behavior in the United States. One recent study found that immigrants of New York City, the city with the highest immigrant count in the country, are just as likely and sometimes more likely to engage in environmental behaviors than native-born residents (Pfeffer and Stycos, 2002). However, other studies found no differences in environmental beliefs among immigrants compared to the majority (e.g., Lovelock et al., 2012). Follow-up studies would be necessary to investigate the replicability of this study among different immigrant ethnic groups and within different regions of the country. Future findings may reveal a country of origin effect for immigrant environmental behaviors. After all, cultural biases toward the environment exists based on different society types. For example, individualistic cultures tend to have less environmental engagement, while hierarchical and egalitarian cultures tend to have more (for a review, see Price et al., 2014). Thus, future research should pursue evidence on how people who possess different cultural orientations or countries of origin translate environmentalism into the American cultural context.

## Investigating Local Economics and Sociocultural Factors

Future research needs to investigate external factors that influence ethnic minorities' environmental behaviors, such as economic and sociocultural factors (Kollmuss and Agyeman, 2002; Gifford and Nilsson, 2014), rather than “controlling” the factors (an exception see Schuldt and Pearson, 2016). Economic status plays a large role in environmental behavior, effecting an individual's amount of disposable income and amount of exposure to pollutant. Pro-environmental activists across nations tend to be of higher socioeconomic status. There is a strong association between socioeconomic status and pro-environmentalism in high-income and developed regions, and a slight association in lower-income and developing regions (Pampel, 2014). The greater association in high-income countries can be explained through a willingness to pay. A fundamental shift may occur when people no longer need to spend time and resources meeting their basic needs (e.g., income and property). Instead, people will be able to allocate more funds to addressing environmental issues (e.g., purchasing environmentally friendly products and organic foods) (Jones and Dunlap, 1992; Gifford and Nilsson, 2014). Opposing research argues that wealthy individuals (with agency and power) are more likely to dismiss environmental concerns due to increased access to unpolluted resources in their daily lives (Franzen, 2003; Bickerstaff, 2004). For example, wealthier individuals might have a lower risk perception of air pollution because they can afford to live in less urban, industrialized areas.

Sociocultural factors affect environmental action as well. Past research has indicated that age, cohort, political orientations, and educational levels correlate with environmental concern and behaviors (Gifford and Nilsson, 2014). For example, age negatively predicts environmental concern (Barr, 2007), where recent generations tend to be more concerned about the environment (Jones and Dunlap, 1992) possibly a result of the increased prevalence of environmental initiatives or exposure to environmental issues through social media. Education on environmental issues as well as science literacy, are found to positively correlate with climate change risk perception, meaning that individuals who are more adept at interpreting the scientific, environmental literature are more likely to see climate change as a problem (Leiserowitz, 2006). One recent study has examined the political ideology in relation to climate change (Schuldt and Pearson, 2016). They found that political ideology was less predictive of environmental beliefs among members of ethnic minorities than White in the U.S. Thus, future studies should investigate the interactions of age, SES, levels of education, and political views as potential predictors of environmental concerns and behaviors in an analysis of specified ethnic groups to provide deeper insight into potential mitigating factors.

## Recognizing Mixed-Methods Methodology

It is generally the case that quantitative analysis is used as the predominant framework in the field of Environmental Psychology. However, the quantitative analysis does not provide a comprehensive explanation addressing the complexity of the human experience. A mixed-methods approach is a strong option to consider for future research because it employs qualitative methodologies to enrich the meaning of the quantitative data (Scharf and Mayseless, 2011). This type of research design searches for different perspectives among individuals under the premise that embracing their personal, cultural, and historical experiences may shape their environmental worldviews (Pratt and Matsuba, 2018). The mixed-methods approach can provide a fruitful ground to investigate motivations of either pro-environmental or anti-environmental behaviors without biasing answers (e.g., moderacy and extremity biases, acquiescence bias, reference-group effect) by providing any preconceived notions (Pratt and Matsuba, 2018). In the following section, two specific approaches of mixed methodology are suggested in the field of environmental psychology.

Triangulation mixed-method design (Creswell and Plano Clark, 2007) has been recently used in the field of environmental psychology. In this approach, researchers collect quantitative data to examine expected relationships as well as parallel qualitative data to address the same research question. Both quantitative and qualitative data are used to confirm, cross-validate, and support findings. This approach also offers researchers the flexibility to convert qualitative data into numerical data (Creswell and Plano Clark, 2007). A recent study conducted by Jia et al. (2016) applied this triangulation mixed-method design. In their study, a positive relationship between generative concern and environmental identity was established by a set of questionnaires. This quantitative result was supported by qualitative interviews of environmental narrative identity. They found that participants with high levels of generative concern tended to tell more meaningful, vivid, and impactful environmental narratives. In addition, different aspects of generative concerns (feeling of empowered to help environment; having children as a focus for crystalizing environment; and passing family traditions in environmental activities) were expressed via environmental narratives (Jia et al., 2015).

In contrast to the triangulation design, the explanatory design offers a two-step approach: a primary focus on quantitative analyses of the study and follow-up qualitative data in an effort to provide a comprehensive explanation for the quantitative analyses (Creswell and Plano Clark, 2007). For example, Jia et al. (2017) conducted two studies to exam how moral identity (both value and motivation) related to levels of environmental engagement. In study one (Jia, 2017), they found that self-transcendent moral values measured using a set of questionnaires (Krettenauer et al., 2016) positively predicted environmental engagement. However, researchers have raised concerns that values do not significantly contribute to the motivation of why people involve in pro-environmental behaviors (Kaiser, 2006; De Groot and Steg, 2007). Qualitative analyses in the follow-up study further explained the motivational factors driving environmental involvements. By using thematic analyses, they found three self-transcendent themes (1. Concern for other species; 2. Vigilance for the environment; 3. Moral emotions toward environmentally irresponsible others) that motivated a group of environmental activists' engagement. Thus, researchers should be encouraged to design mixed-methods studies that provoke ethnic minority participants' perceptions, beliefs, and opinions on environmental issues and capture the rich and complex cultural messages in a meaningful context.

## Conclusion

In conclusion, globalization and migration have resulted in a growing need to understand ethnic minority members' environmental engagement in a heterogeneous society. However, the literature provides no clear indication of how ethnic minority groups might respond to the environmental issue. This controversy calls for new considerations on theories and methodologies in future research. Bronfenbrenner (1979) ecological model may provide insights on the topic considering individual, social, and cultural levels of analyses. Researchers should favor a holistic approach that evaluates individual, social, cultural, economic, and political influencers. Researchers should consider sociocultural and socioeconomic factors and spans multiple levels of cultural diversity and orientation. Statistical methodologies should strive to use a mixed-methods approach that integrates both quantitative and qualitative data. These strategies can provide researchers with more insight into motivations behind ethnic groups′ environmental concerns and behaviors in the United States. Together, this article opens a further inquiry in contexts of concerns and involvements among ethnic groups such as senses of personal agency and social construction in addressing environmental issues.

## Author Contributions

VM wrote an initial draft. AD added a large section of the manuscript. NR provided an initial literature search and summary. DC wrote one section in the initial draft. FJ conceptualized the framework, wrote and revised a significant portion of the manuscript, and advised the writing process.

### Conflict of Interest Statement

The authors declare that the research was conducted in the absence of any commercial or financial relationships that could be construed as a potential conflict of interest.
